# Verification of the Conditions for Determination of Antioxidant Activity by ABTS and DPPH Assays—A Practical Approach

**DOI:** 10.3390/molecules27010050

**Published:** 2021-12-22

**Authors:** Rafał Wołosiak, Beata Drużyńska, Dorota Derewiaka, Małgorzata Piecyk, Ewa Majewska, Marta Ciecierska, Elwira Worobiej, Paulina Pakosz

**Affiliations:** Department of Food Technology and Assessment, Division of Food Quality Assessment, Institute of Food Sciences, Warsaw University of Life Sciences (WULS-SGGW), 159 Nowoursynowska Street, 02-776 Warsaw, Poland; beata_druzynska@sggw.edu.pl (B.D.); dorota_derewiaka@sggw.edu.pl (D.D.); malgorzata_piecyk@sggw.edu.pl (M.P.); ewa_majewska1@sggw.edu.pl (E.M.); marta_ciecierska@sggw.edu.pl (M.C.); elwira_worobiej@sggw.edu.pl (E.W.); paulina_pakosz@sggw.edu.pl (P.P.)

**Keywords:** reaction solvent, reaction time, assay comparison, IC_50_, TEAC, phenolic, polyphenol, peptide, glutathione, amino acid, ascorbic acid, tocopherol

## Abstract

The ABTS and DPPH methods are among the most popular assays of antioxidant activity determination. Attempts to adapt them to different analytes and the search for the highest values of antioxidant activity has resulted in a large variety of assay conditions to be presented in the literature, including the way the measurement is made. This makes it difficult to relate the results to real oxidation systems, and often makes it impossible to compare them. Such a comparison is limited in advance by the use of stable radicals that do not exist in nature and that react differently from those generated in food or in vivo. Therefore, it is important to introduce measures aimed at standardizing the conditions of the activity assay, including reaction time and several reaction environments suitable for testing different groups of compounds. In this study, we used natural antioxidants of various structures: phenolic acids, flavonoids, peptides and corresponding amino acids, ascorbic acid and α-tocopherol, and also synthetic analogues of selected compounds. The curves of dependence of the measured absorbance on the concentration of antioxidants were described, the ranges of linearity were determined, and the value of the error made when reading in various ranges of dependencies was estimated. We also determined and compared the activity values using two popular methods (IC_50_ and TEAC), taking into account different environments and reaction times. Based on the collected data, recommendations were formulated regarding the reaction conditions adapted to the studies of individual groups of antioxidants, and unified reaction times were proposed. Taking into account the state before reaching the equilibrium of antioxidants reacting in a complex manner, this approach may introduce a simplified reference to the competing reaction that occurs in reality.

## 1. Introduction

Aerobic cellular respiration, as adopted by highly organized organisms living on Earth, provides much energy for their functioning, but also causes side effects from metabolism in the form of free radicals. In defending themselves against the damaging effects of radical presence, living organisms use exogenous and endogenous antioxidants. These compounds play a very important metabolic role, and studies on the antioxidant activity of substances extracted from food or biological material has been the subject of researchers’ interest for many years. It seems that these studies will not lose their relevance given the progressive discoveries regarding the role of antioxidants not only in ensuring food stability or the metabolic balance of organisms, but also in the course of diseases and aging processes. The activity of antioxidants can be tested using various analytical techniques relating to the different properties of the test compounds on the one hand, and to the different stages of the oxidative process and a different understanding of the role of antioxidants on the other [1,2,3]. Research with the use of stable free radicals ABTS and DPPH, which can be determined spectrophotometrically and discolored as a result of deactivation, is particularly popular. However, their popularity, ease of use and susceptibility to modification have resulted in the appearance of many publications in which, because of the different versions of the assays used, the results are not comparable.

A proposal for the use of 2,2’-azino-bis-3-ethylbenzothiazoline-6-sulfonic acid (ABTS) to generate a stable radical cation by reaction with an oxidant and the measurement of the “total antioxidant capacity” of, i.a., body fluids appeared in 1993 [4]. Initially, the method was presented as additive and consisted in the continuous formation of ABTS^•+^ from the substrate present in the reaction environment as a result of the action of hydrogen peroxide in a reaction catalyzed by the addition of metmyoglobin. The authors also introduced Trolox, a synthetic analog of α-tocopherol with increased solubility in aqueous solutions, as a standard antioxidant. The reaction was carried out in PBS (phosphate buffered saline) [5]. Due to the possibility of overestimation of the results because of the possible reaction of some antioxidants with the substrates or with the intermediate products, a new version of the method was developed in which the radicals were previously produced from ABTS by direct reaction with potassium persulfate (post-additive method) for at least 6 h. Then radicals were diluted with PBS or ethanol to obtain an absorbance of 0.7 and were mixed with the sample. The determination consisted in measuring the residual radicals after the assumed reaction time (6 min). In the publication on the new version of the method, the authors showed the possibility of its application in relation to both food components and blood plasma [6].

Another, currently the most popular, antiradical activity assay, which uses the deactivation of the synthetic, stable DPPH radical (2,2’-diphenyl-1-picrylhydrazyl), has been popularized mainly because of the work of Brand-Williams et al. [7]. The authors proposed the use of a methanolic radical solution (DPPH^•^ are available as ready-made radicals—the simplicity of their use probably has an impact on their popularity) to study the antioxidant activity of food ingredients by measuring the absorbance of radicals remaining in the reaction environment. In this study and others [8], the researchers used the measurement of residual radicals until reaching an equilibrium plateau. The time needed for some compounds to react fully was even many hours. In further works, the simplification of the procedure was popularized by setting the assumed reaction time, usually by returning to 30 min from the first study with these radicals [9]. The use of units of relative activity (% of radical deactivation) or conversions based on the activity of a standard antioxidant, usually Trolox or ascorbic acid, has also become widespread instead of calculations based on reaction kinetics (e.g., the time needed to deactivate half of the radicals assuming a standard ratio and concentration of antioxidant and radical) [10,11].

Due to the constraints of the constant reaction time, the possibility of determining the “total antioxidant capacity” of the samples is questioned. Rather, we obtained the possibility of a relative evaluation (“ranking”) of the antioxidant activity. However, this does not have to be treated as a disadvantage, because the determination of the total antioxidant capacity of tested antioxidants by simple in vitro methods is of no significance in relation to their actual effectiveness in vivo or in situ (in food), where all potentially active functional groups will not have a chance to react completely [12]. Steric accessibility also plays an important role in reactivity: molecules without large substituents that are hindered have better access to the radical, which is of particular importance in relation to DPPH^•^. Radicals are also deactivated through various mechanisms, the most basic of which are SET (electron transfer) and HAT (hydrogen transfer). However, both ways can be used simultaneously for deactivation, depending, e.g., on the characteristics of the antioxidant and the reaction environment. Some antioxidants (especially those with a simple structure and one reactive group, such as ascorbic acid) reach equilibrium in less than 1 min, but other compounds, with more complex structures and potentially with multistage action, require longer reaction times. This influences the useful absorbance range, which is limited by the obtained non-linear activity dependencies on the concentration of tested substances, which are additionally influenced by the composition of the reaction environment [13].

The aim of this study was to determine the applicability of simple and common methods of testing antioxidant activity (against ABTS^•+^ and DPPH^•^) to various antioxidants present in food and biological systems, as well as to determine the linearity of the dependence of absorbance on the concentration of antioxidants and the influence of changing reaction conditions on the results obtained. The aim was also to determine reliable ways of reading the results and to formulate practical guidelines for selecting reaction conditions to ensure uniformity of testing and comparability of results.

Several model groups of antioxidants and their equimolar mixtures were used in the study. The selection was based on the universality of the compounds in food and their antioxidant properties. The research used phenolic compounds, antioxidant peptides and their amino acids mostly responsible for the activity, ascorbic acid and α-tocopherol. The work also utilized synthetic antioxidant analogs, used as food additives or analytical standard (Trolox).

## 2. Results and Discussion

### 2.1. Dependence of the Measured Absorbance on the Concentration of Antioxidant

The ABTS method is less dependent on the type of solvent used to prepare the solutions of the tested antioxidants, because the volume of this solvent (keeping the original proportions) is 100 times smaller than the volume of the radical solution. Hence, the solvent used to prepare the radical solution should largely determine the properties of the reaction mixture. In the research, the method is widespread in two versions: with ethanol-diluted radicals for the study of non-polar or less polar substances, and with radicals diluted with PBS (pH 7.4) for the study of mainly polar substances while simulating the physiological environment. However, other modifications of the method can also be found. We have also chosen an acetate buffer with a pH characteristic for food products—already used for simulating the composition of wine [14]. 

We investigated the solutions of model antioxidants in 50% acetone and methanol (which allowed the use of all tested antioxidants) in the assay with ethanol-diluted radicals. Tests were also carried out in buffers pH 3.6 and pH 7.4. In line with the original methodology and in order to simplify the composition of the reaction mixture, the same buffers were used to dilute the radicals and prepare solutions of the test substances. Selected versions of the test systems (the analysis of polar substances, for which this method provides greater application possibilities) were used not only in the standard reaction time (6 min), but also in extended time (30 min). The obtained linear or non-linear functions of the dependence of absorbance on the test substance concentration are presented in Table 1.

Among the four tested natural phenolic compounds, only in the case of gallic acid were linear dependencies found after standard reaction time in the entire absorbance range recommended in the original publication. The effects of gallic acid are similar to those of its synthetic derivative, propyl gallate. After the use of quercetin, the linearity of the relationship was limited, and no linear relationships were found after the use of ferulic acid and catechin. After extending the reaction time to 30 min, linear results were already noted for all phenols except ferulic acid, which confirms that the deficiencies in linearity result from the different reaction rates of individual phenols with ABTS^•+^ [12]. By examining the peptides and the corresponding amino acids, different effects of the use of imidazole compounds, carnosine and histidine (only a trace of carnosine activity, mainly in ethanol, tested up to 80 μM in the reaction mixture) were noted compared to the active sulfhydryl antioxidants. Their application to radicals in ethanol, however, resulted in a lack or a limitation of the linear course with the standard and the extended reaction time. These compounds behaved better in buffers at the standard reaction time—cysteine at pH 3.6, and both compounds at pH 7.4, gave linear relationships. Surprisingly, linearity was lost with extended time. Zheng et al. [15] proposed a two-step mechanism for the ABTS^•+^ reaction with amino acids: a fast first step followed by a slow step of re-reacting the single-electron-oxidized amino acid with a new radical molecule. This may explain the limitations in linearity observed in the study at higher concentrations and the loss of linearity with prolonged reaction times. In contrast to the compounds described above, ascorbic acid showed a good linear relationship between activity and concentration over the entire absorbance range tested, regardless of the solvent used. The same effect resulted from the use of the antioxidant standard in the ABTS assay, Trolox, as well as ascorbyl palmitate and tocopherol (last two compounds in methanol). The lack of tocopherol acetate activity due to blocking of the reactive phenolic group of α-tocopherol was also confirmed in the assay. 

Small and fast-reacting molecules of antioxidants, including compounds with a single phenolic group, gave linear responses with increasing concentration, while the more complex structure favoring the formation of adducts, the presence of additional phenolic rings (flavonoids), and many hydroxyl groups of different reactivity (flavonoids, phenolic acids), caused the reaction to take more time to reach equilibrium—hence the curves became non-linear [12]. These authors reported a particularly large impact on the linearity of the reaction in the case of ferulic acid, glutathione and quercetin, which is consistent with the data collected in this study.

In the case of the mixture of phenolic compounds tested in ethanol, linearity limitations were found after both reaction times, while amine compounds showed a linear relationship with prolonged reaction times (Table 1). A similar effect was observed after the use of the mixture of polar compounds, but the limitation of linearity with a shorter reaction time was consistent with that of phenolic compounds, and not of amine compounds. Phenolic compounds most likely caused a similar effect of the mixture of all compounds applicable in methanol (phenolic, ascorbic acid and α-tocopherol, possible, e.g., as a result of extraction of dehydrated material with pure methanol), despite the linear characteristics of the last two compounds in this version of the experiment. Peptides and amino acids showed non-linear relationships at pH 3.6 and linear at pH 7.4, and the combination of all natural polar substances (gallic acid, peptides, amino acids, and ascorbic acid) allowed only linear relationships to be obtained in both buffers.

Contrary to the ABTS assay, the solvent used for the preparation of antioxidant solutions in the DPPH method was of fundamental importance for the properties of the reaction mixture due to its much larger volume compared to the volume of radical solution—4:1. The large volume for the sample also makes it possible to introduce the right number of antioxidants in more diluted extracts. In the research, the DPPH radical is generally dissolved in methanol, but other solvents were also used in the study and resulted in a uniform, fully aprotic reaction environment. This made it possible to test the method more widely in determining the effects of less polar substances. To prepare the model antioxidant solutions, we used 50% acetone, 70% acetone, 100% acetone (with the radical prepared in acetone as well); 50% methanol, 80% methanol, 100% methanol; ethyl acetate (with the radical prepared in ethyl acetate as well); hexane (with the radical prepared in hexane or acetone) and dichloromethane (with the radical prepared in methanol or dichloromethane). In two test systems (50% acetone + methanol and methanol + methanol), the standard reaction time (30 min) was supported by two other reaction times: shorter (10 min) and extended (120 min). The decision was made to ensure that all compounds could be tested at different reaction times. The lower limit of the absorbance working range for the DPPH method was set at 0.1 (compliance with the Lambert–Beer law was confirmed on this absorbance), whereas the upper limit of the absorbance (absorbance of control) depended on the solvents used and the reaction time, and amounted to 1.0–1.2. The obtained linear or non-linear functions of the dependence of absorbance on the test substance concentration are presented in Table 2, Table 3 and Table 4.

In a mostly protic environment, 50% acetone mixed with methanol (4:1), gallic acid and quercetin showed a linear relationship over the full range tested with standard and extended reaction times (Table 2). Ferulic acid showed linearity limitations (decreasing with the reaction time). In the case of catechin, linearity limitations were noted initially, but at longer times completely non-linear relationships were noted. The synthetic derivative of gallic acid, propyl gallate, again behaved in a manner similar to gallic acid (only linear relationships in this environment). Increasing the acetone concentration to 70% (and thus changing the environment to a polar, but mostly aprotic environment) limited the linearity of all phenols. A further increase in acetone concentration resulted in non-linear relationships of natural phenolic acids, and the use of a fully aprotic environment (with radicals given in acetone) resulted in non-linear relationships of all natural phenols (or inability to determine a function in the case of quercetin). The same phenomenon was observed in a fully aprotic environment of ethyl acetate (Table 4), while the introduction of methanol therein as a radical solvent gave linear or linear-with-limitations functions (except for ferulic acid). The use of the tested phenolic compounds in the medium of 50% methanol with the addition of radicals in methanol gave similar results as in the case of 50% acetone (except for the lack of quercetin solubility, Table 2). The increase in the concentration of the solvent used for sample preparation to 80% methanol had similar effects as the increase in acetone concentration (except for the good linearity of quercetin). However, the use of pure methanol at the standard time of 30 min resulted in a good linearity for gallic acid and catechin, with slight limitations on the linearity of quercetin and very large limitations on that of ferulic acid. Only with quercetin, the extension of the reaction time worked well in terms of linearity. Propyl gallate behaved in a strictly linear manner in all methanol systems.

The solubility of peptides and amino acids in the systems used in the DPPH assay was very limited. Glutathione tested in acetone solutions gave non-linear relationships, regardless of the reaction time, and cysteine gave linear relationships (Table 2). The same was noted in 50% methanol (Table 3). At longer reaction times, carnosine and histidine showed a trace reactivity when used in 50% acetone, while in 50% methanol only histidine showed a trace reactivity. Ascorbic acid could be used in aqueous acetone; at 50% concentration it showed both an upper and a lower limit of linearity, as did its palmitic ester when given in pure acetone (it could be tested at an acetone concentration of at least 70%). Analogous bilateral linearity limitations also occurred for ascorbic acid in 50% methanol and pure methanol in the shortest reaction time. Extending the reaction time to 30 min resulted in the elimination of the upper limit of linearity, and a further extension of the reaction time to 120 min also eliminated the lower limit. Ascorbyl palmitate used in pure methanol gave only linear curves, as in ethyl acetate (Table 4). Tocopherol tested in acetone showed a slight limitation of linearity when methanol was introduced into the system. This was similar to Trolox, which in all other acetone systems gave strictly linear relationships. Both analogs were already strictly linear in the methanol-based systems. Slight problems with linearity reappeared in ethyl acetate. The course of the dependence of absorbance on the concentration of α-tocopherol in the case of traditional non-polar solvents, hexane and dichloromethane, was already strictly linear. Tocopherol acetate showed traces of activity in acetone-containing systems despite the blocked phenolic group (tested to 2.5 mM in the reaction mixture).

The mixture of phenols in most of the tested experimental systems showed limitations of linearity applied to DPPH radicals (Table 2 and Table 3). In an acetone solution, they increased with increasing reaction time, in contrast to methanol. The mixture of polar compounds had limitations in linearity similar to those of the mixture of phenols. The mixture of all natural compounds applicable to methanol showed limitations in linearity after each reaction time, which is understandable considering that, of the compounds used, only gallic acid and tocopherol were free of them.

On the basis of the experiments conducted, it can be concluded that the ABTS assay provides much greater possibilities for research on bioactive amine compounds, due to the greater polarity of the environment and the solubility of peptides in the solvents used. However, significant limitations in linearity are to be expected when considering the use of a wide absorbance range. Phenolic compounds can be tested in both methods, but the DPPH method offers much wider possibilities of changing the reaction environment (which, however, is not conducive to the standardization of the tests). One can also expect non-linear dependencies or limitations in linearity: regardless of the predominant class of compounds, these features will be exhibited by both phenolic acids and polyphenols. Ascorbic acid is completely immune to linearity problems in an ABTS assay that always contains even a small amount of water. In order to avoid linearity problems in the DPPH method, acetone and methanol solutions should be used at the intermediate concentrations typically used for phenol extraction. On the other hand, it should be taken into account that this is an environment that significantly limits the stability of ascorbic acid and may limit the activity of amine compounds. The DPPH assay is not naturally predisposed to buffering the solution—there are problems with the formation of iridescence in the presence of amine compounds—and will not withstand acidification of the environment with a strong acid (immediate free radical deactivation). Thus, antioxidant extracts in which ascorbic acid or peptides are of significant quantitative importance should be tested by the ABTS method. Contrary to that, the DPPH method offers much greater possibilities for studying non-polar compounds. Trolox behaves very linearly in all experimental systems, which confirms its suitability as an antioxidant standard. The same is true of other synthetic antioxidants.

### 2.2. Activity Expressed by IC_50_ Values

Table 5 shows the IC_50_ values obtained by applying different variants of the ABTS method. At the standard reaction time, most of the tested antioxidants and their mixtures had the highest activity against ethanol-diluted radicals, dissolved in 50% acetone or methanol (the reaction mixture contained mainly ethanol). The trace activity of carnosine did not allow it to be expressed in the category of 50% inactivation of radicals. The use of an environment with pH 3.6 (the acidic pH found in foodstuffs) caused in almost all cases a clear reduction of the activity. At the physiological pH of the reaction medium, medium activity was found, with the exception of gallic acid and cysteine, which were the most active (cysteine showed the reverse sequence compared to most of the tested compounds: pH 7.4 > pH 3.6 > ethanol). The pH of the environment has a great influence on the activity of amino acids, and thus on peptides [15]. The smallest differences in activity when changing the parameters of the experiment were observed for the compounds with the highest activity: gallic acid and its ester, ascorbic acid and Trolox. The even activity of the latter (11–12 µM after 6 min) ensured a uniform reference point as the antioxidant standard. The decreased activity of ascorbic acid, gallic acid and glutathione in the acidic environment with practically unchanged activity of Trolox has also been (at pH 6.5) described in the literature [16], as well as the lower activities of phenolic antioxidants [17]. Taking into account the values obtained in a system diluted with ethanol for various substances at a standard reaction time, it can be concluded that phenolic compounds were the most active in this experiment, followed by glutathione, then Trolox, α-tocopherol, ascorbic acid and ascorbyl palmitate, with the lowest activity shown for cysteine. After extending the reaction time from 6 to 30 min, there was no change in the activity of rapidly reacting compounds (ascorbic acid and Trolox) and there was an increase in the activity of the remaining compounds. The change in activity was usually below 50%, except for the antioxidant with the lowest activity, i.e., cysteine. It is known from the literature that peptides and amino acids do not reach reaction equilibrium immediately, but only after much longer times than the standard 6 min. It has been estimated to be at pH 7.4 for about 30 min [15].

The activities calculated in the DPPH method as the concentration required for 50% radical inactivation were generally lower compared to those in the ABTS method (Table 6, Table 7 and Table 8). The activities of natural compounds (phenolic, glutathione, ascorbic acid and α-tocopherol) obtained in aqueous and pure acetone after a standard reaction time clearly decreased along with an increase in the aprotic component. This was particularly evident when other solvents were completely eliminated and especially for the less active antioxidants (glutathione, ferulic acid and catechin). The effect was weaker with Trolox and propyl gallate, and not strict for ascorbyl palmitate. Taking into account the activities obtained in 50% acetone and 100% acetone, their order is similar to that in the ABTS method, except for amine compounds: phenolic compounds (especially gallic acid and quercetin) ≥ Trolox > ascorbic acid ≈ ascorbyl palmitate_ACN_ = Trolox_ACN_ = a-tocopherol_ACN_ > cysteine > glutathione. This observation—the very low activity of glutathione and cysteine—demonstrates once again the need to test such compounds by the ABTS method in buffers.

The activities obtained in 50% methanol were low and similar to those obtained in 50% acetone (Table 7). After increasing the concentration of methanol used to prepare the sample to 80%, they also decreased (in the case of some phenols, even twice), while after using pure methanol, the activity increased again. Only in this solvent was the activity of Trolox identical to the activity in the ABTS method, while at the same time twice as high as the activity of α-tocopherol and ascorbyl palmitate and three times as high as that of ascorbic acid. The phenolic compounds had activities intermediate between those obtained with 50% methanol and 80% methanol. The summary of the activity read under different conditions presented in this paper clearly shows the great importance of the appropriate selection of the reaction environment for the correct interpretation of the results obtained and their accurate reference to the data in the literature. For example, the similar activity of quercetin and propyl gallate used in equal molar concentrations against DPPH radicals, emphasized by Decker et al. [18], corresponds to the data obtained in this paper in methanol, 80% methanol and 50% acetone, but definitely not in 70% and 100% acetone.

The use of ethyl acetate as a sample solvent in combination with radicals in methanol followed by complete replacement of the methanol with ethyl acetate had very similar effects as the use of acetone (Table 8). The only exception was the activity of Trolox—it was similar in the case of methanol and more than seven times lower when using a completely aprotic environment of ethyl acetate with only a slight reduction in pure acetone. On the other hand, the activity of α-tocopherol in all systems based on acetone, ethyl acetate, dichloromethane and hexane underwent very few changes, while in methanol it was about 30% higher. This suggests that a DPPH assay can be used to study non-polar antioxidants. To compare the activity under different conditions—polar protic (methanol), polar aprotic (acetone) and apolar (hexane, dichloromethane)—different solvents may be used so long as the solubility of the sample allows it. The use of apolar solvents precludes the standardization of results with Trolox, but α-tocopherol could become such a standard under these conditions.

The change of reaction time in 50% acetone (Table 6) and methanol (Table 7) was generally more effective in the first period considered (from 10 to 30 min) for both phenolic and sulfhydryl antioxidants. For typical fast-acting antioxidants (ascorbic acid, ascorbyl palmitate, Trolox, α-tocopherol), the prolonged reaction time did not affect the activity or the effect was very insignificant (ascorbyl palmitate). The results obtained in the study confirm the observations of changes in antioxidant activity over time (10–120 min) made by Ozgen et al. [19], in which no significant changes in the activity of rapidly reacting compounds, ascorbic acid, Trolox and also gallic acid, were found, in addition to relatively small changes in the activity of quercetin (mainly in the range of 10–30 min) and significant differences in the activity of other phenolic acids.

This indicates a well-chosen reaction time of 30 min, which allows taking into account the different effects of antioxidants of different effectiveness (reactivity) without reaching or approaching the equilibrium state for all compounds which would not have a real reference to the in situ situation in food or the in vivo situation, where an account of the full antioxidant capacity of samples determined in vitro is not possible.

### 2.3. Activity Expressed by TEAC Values

Another way to express the activity of antioxidants is by relating them to the activity of the Trolox standard. In the case of samples of unknown composition, this is done by reading the amount of Trolox (usually mg or μmol) corresponding to the activity of the tested extract and the conversion to the specified amount of the initial sample. However, performing a reading at different the relative activity of the test extracts can easily cause falsified results. For the research carried out in the study, it was possible to compare the IC_50_ parameter of the tested antioxidants or their mixtures to the IC_50_ of Trolox, i.e., to perform a uniform reading with 50% radical deactivation. Moreover, it was also possible to determine the number of Trolox molecules with an activity corresponding to that of an antioxidant molecule applied to different radicals and under different conditions. Taking a reading of the antioxidant activity at any degree of radical deactivation, and then expressing it in Trolox units, in order to convert it to a certain mass or volume of the original sample, requires the assumption of a linear dependence of the antioxidant effect on the number of antioxidants tested. As shown in the study, these relationships are often non-linear. The non-linear curves obtained allowed for the visualization of the magnitude of the reading error at activities significantly deviating from 50%. For such a comparison the values of IC_80_ (80% inactivation of the radicals, A = 0.2 × A_contr_) and IC_20_ (20% deactivation of the radicals, A = 0.8 × A_contr_) were determined and then referred to the respective Trolox concentrations to obtain the TEAC values. The results are shown in Table 9, Table 10, Table 11, Table 12 and Table 13.

Due to the very even activity of Trolox, the activities expressed in the ABTS method in TEAC units reflect the IC_50_ values, while on the contrary, the TEAC values are directly proportional to the antioxidant activity, which facilitates the analysis of the results. When radicals in the measurement system were diluted with ethanol, the ascorbic acid molecule acted identically to the molecule of its synthetic derivative and α-tocopherol—and all exhibit comparable activity to Trolox (Table 9). The activity of the glutathione molecule is similar to that of Trolox, while the activity of phenolic compounds exceeds Trolox by 1.5 to over 3 times. The activity of the synthetic gallic acid derivative, however, is smaller than that of the precursor molecule. Indicatively, the activity of carnosine (due to the possibility of reading from the Trolox standard curve, which was not possible in the case of the IC_50_) was approximately one hundred times lower than the activity of Trolox.

Activity reading errors occur at higher antioxidant concentrations, but are particularly large in general at low concentrations (IC_20_), where the reading error due to non-linearity is added to the error due to low concentration conversion. Among the mixtures of antioxidants, the greatest errors were possible in the case of the mixture of phenolic compounds and mixtures of various compounds. The use of buffers modified the TEAC values in line with the trends described above, but the errors that could be determined were much smaller (Table 10). The TEAC values obtained in the study are very similar to the literature data for gallic and ferulic acid, catechin, ascorbic acid, glutathione and tocopherol—both in the additive and in the post-additive version of the method [4,5,6,20]. The reported values for quercetin are however very different in the ABTS method, both being consistent with the values determined in this study and even twice as high [21].

In the DPPH assay, a molecule of ferulic acid tested in aqueous acetone behaved as only about 0.5 of the Trolox molecule did, and the most potent gallic acid behaved similarly to fewer than 3 Trolox molecules (Table 11). The activity of glutathione did not exceed 20% of Trolox molecule activity, and ascorbic acid did not exceed 80% of this value. The determination of errors in the reading of ascorbic acid activity was not possible due to the lack of any non-linear model to fit the data series obtained. Apart from the quercetin in 80% ethanol, the erroneous readings determined are smaller to those obtained with the ABTS method. This also applies to the tested antioxidant mixtures. The TEAC values obtained in aqueous methanol (Table 12) are greater than those obtained with a similar concentration of acetone solution, but there is also the possibility of generating larger errors. However, mixtures of antioxidants were more resistant to these errors than individual substances. The TEACs calculated on the basis of the data collected in methanol are clearly the lowest (Table 13). The value for the mixture of phenolic compounds is over four times lower than in 50% methanol and over three times lower than in acetone solutions. Deactivation of DPPH^•^ can occur via single electron transfer (SET) and hydrogen atom transfer (HAT), the latter being the preferred mechanism if possible (as opposed to ABTS^•+^). The presence of methanol binds hydrogen atoms and greatly reduces HAT, but the presence of water facilitates this transfer [12]. 

Most phenolic compounds again showed unusually low activities in ethyl acetate with radicals in methanol, but the activities of ascorbyl palmitate and α-tocopherol were identical to those of Trolox (Table 13). The TEAC value obtained for glutathione in the DPPH method was several times lower than in ABTS, but a comparable value was obtained in the case of cysteine. Since the standard curve of Trolox solutions can potentially be used to express the activity results as TEAC, the approximate values of carnosine and histidine activities were calculated for comparison purposes, similarly to the ABTS method. These values were ~0.0007 and ~0.0004, respectively; they were therefore three orders of magnitude lower than those specified for the remaining amine compounds.

To summarize, the highest activity values of phenolic compounds after their standardization with reference to Trolox in the DPPH assay were obtained in aqueous methanol. The same was true for the activity of glutathione, cysteine and ascorbic acid, although, as mentioned earlier, the ABTS assay provided a much better system for their testing. It is easy to ensure such a reaction environment with a 4:1 ratio of the sample volume to the radical solution volume, because even with weakly acting or diluted antioxidant extracts, the use of 4 mL for the sample introduction seems unrealistic. In turn, for the study of non-polar and low-polar compounds, assuming the results are to be related to Trolox activity, a better reaction environment than pure methanol is ensured by its combination with ethyl acetate.

This study did not investigate the influence of the reaction temperature on the results obtained, although there is no doubt that such an influence exists. It is especially large in the case of significant temperature deviations from room temperature and slow-reacting antioxidants. However, the use of the calibration curve from Trolox solutions as a reference for the measured activity of the samples will allow to some extent for the reduction of the effect of fluctuations in the measurement temperature, because the absorbance of the reference and the control should be recorded under the same conditions.

## 3. Materials and Methods

### 3.1. Materials

The research materials selected were polar compounds with potential antioxidant activity and different active functional groups: phenolic acids (gallic acid and ferulic acid); flavonoids ((±)catechin and quercetin); L-ascorbic acid; sulfhydryl compounds (glutathione and L-cysteine); and imidazole ring compounds (carnosine and L-histidine). The research material also included one non-polar antioxidant, α-tocopherol, and common synthetic derivatives or analogs of selected antioxidants, namely, propyl gallate, ascorbyl palmitate, Trolox (6-hydroxy-2,5,7,8-tetramethylchroman-2-carboxylic acid) and (for comparison purposes) α-tocopherol ester with blocked phenolic group (tocopheryl acetate). These compounds were tested separately or (in chosen experiments) in equimolar mixtures of natural phenolic compounds, amino acids and peptides, applicable polar compounds or all natural antioxidants applicable in the solvent. All tested compounds were purchased from Sigma-Aldrich (Poland). All other chemicals were of analytical grade.

Two buffers were used in the experiments: 300 mM acetate buffer pH 3.6, simulating an acidic reaction medium typical for food products, as well as PBS: 10 mM sodium phosphate buffer pH 7.4 containing 138 mM sodium chloride, used to simulate the physiological environment of human body fluids.

### 3.2. Methods

#### 3.2.1. Activity against ABTS Radical Cations

ABTS^•+^ were generated according to the original method of Re et al. [6] by mixing equal volumes of substrate solution (2,2′-azino-bis(3-ethylbenzothiazoline-6-sulfonic acid, 7 mM) and oxidant (potassium persulfate, 2.45 mM); the reaction was carried out in the dark for 12 h. After this time, the radical solution obtained was diluted with ethanol or buffers of pH 3.6 and 7.4 to an absorbance of 0.70 ± 0.10 measured at 734 nm. Test compound solutions (40 µL) were collected in test tubes, and then 4 mL of the radical solution was added. The reaction was performed for a standard time (6 min), and in some experiments also for a prolonged time (30 min), after which the absorbance was again measured at 734 nm. The analyzes were performed in 4 replications. Tested antioxidants were used as the solutions in 50% acetone (6 or 30 min of reaction) and methanol (6 min of reaction) in the case of radicals diluted with ethanol, and in an appropriate buffer against radicals diluted with buffers (6 or 30 min of reaction). The antioxidants and their mixtures were tested at various concentrations (until the absorbance of 0.14 was reached) in order to determine the course of the dependence of the absorbance resulting from the residual radicals after the reaction on the concentration of antioxidants.

#### 3.2.2. Activity against DPPH Stable Radicals

The assay was performed in accordance with Brand-Williams et al. [7] in a modification allowing for an effective change of the prevailing reaction environment: a solution of radicals (0.5 mol/L) in methanol was prepared, then 1 mL of this solution was combined with 4 mL of appropriately diluted antioxidant solutions. In the standard version of the assay, the absorbance was measured at 517 nm after 30 min of reaction [11], and in selected variants also after a shorter (10 min) and a longer (120 min) reaction time. The analyzes were performed in 4 replications. Tested compounds were prepared in 50% aqueous acetone, 70% aqueous acetone, 100% acetone, 50% aqueous methanol, 70% aqueous methanol, 100% methanol, ethyl acetate, dichloromethane and hexane. In chosen experiments, the radical solution was also prepared in other solvents to exclude methanol from the reaction mixture. Concentrations of the antioxidants were adjusted in each experiment to determine the course of the absorbance–concentration curves in the range starting from the absorbance of the control (value depending on the solvent) to the value of 0.1.

#### 3.2.3. Analysis of the Results

Curves of the dependence of the read absorbance on the concentration of antioxidants were determined by regression analysis. Linear regression analyzes were performed using the Statistica Plus 10.0 PL software (α = 0.05). The criterion for the existence of a strict linear relationship was a correlation coefficient amounting to at least −0.99. In the absence of such a relationship, the variables were described by the non-linear function equation with the highest correlation coefficient using the Statgraphics 4.1 software (α = 0.01). Non-linear curves were also applied in the later part of the calculations to determine the potential errors of the obtained activity in the absence of a linear relationship in all or part of the range of tested concentrations, and also if their fit to the tested data series was stricter at α = 0.01 than the corresponding linear curve obtained at α = 0.05.

Based on the absorbance–concentration curves, the activity of antioxidants was calculated as the IC_50_ parameter—the concentration [μmol/L of the reaction mixture] reducing the absorbance of the corresponding control sample by half. They were also converted into TEAC units (Trolox equivalent antioxidant capacity), i.e., as the calculated amount of Trolox molecules with an activity equivalent to 1 antioxidant molecule. In order to estimate the reading error of antioxidant activity for antioxidant solutions causing radical deactivation significantly deviating from 50%, on the basis of the non-linear curves obtained, TEAC values for A = 20% A_contr_ and A = 80% A_contr_ were also determined, referring the readings to the corresponding values for Trolox.

## 4. Conclusions

The standard times for the deactivation of ABTS and DPPH radicals seem to be the most appropriate. On the one hand, they are widely used in the literature, and what is most needed in research on antioxidant activity is the unification of assay conditions. On the other hand, apart from rapidly reacting substances (ascorbic acid, Trolox), these times seem long enough to allow the more reactive phenolic compounds or peptides and amino acids to come closer to equilibrium (as indicated by relatively small gains in activity with longer times), but short enough to take into account the different reaction rates of tested compounds, which actually takes place under real conditions. The postulate of returning to uniform reaction times (6 min for ABTS and 30 min for DPPH) as well as careful selection and limitation of the number of solvents used may replace the complicated procedures that are sometimes proposed, which take into account the kinetics of the antioxidant reaction with radicals. The approach based on the kinetics of reaction is needed when describing the mechanisms of action of individual compounds with reference to their chemical structure, but it does not seem practical for the routine examination of antioxidant mixtures extracted from food or biological samples.

The reaction’s environment often has a greater influence on the results obtained than its time. In the ABTS method, it is best to use ethanol to dilute radicals in the study of phenolic and low-polar compounds. Testing them in buffers is also possible (contrary to the DPPH assay), but due to potential solubility problems (and the possibility of internal structuring, e.g., micelle formation) it would be better to do so while also keeping the ethanol system as a reference. For amine compounds and ascorbic acid, the ABTS method and buffered reaction medium are more suitable. However, the results will be very dependent on the adjusted pH—apart from the traditionally used PBS, we also included acetate buffer pH 3.6, which can simulate the environment of food products (because of both its composition and its pH value) and can also be used in other methods for testing antioxidant activity, making it easier to relate the results. The ABTS method should also be recommended in examining such physiological fluids as saliva, serum, plasma or urine. Trolox is recommended as a uniform standard in the studies of polar compounds. It is most adequate in terms of both the standard physiological pH used in the assay (PBS) and the elimination of potential and solvent-dependent technical complications resulting from protein precipitation and significant mineral salt content.

The DPPH method is most applicable to phenolics and compounds of limited polarity. In the case of phenolic and other polar compounds, aqueous methanol should be introduced into the reaction medium. In the case of low-polarity compounds, ethyl acetate with radicals in methanol is suitable when Trolox is used to standardize the results. In the absence of such an assumption, another non-polar solvent (hexane, dichloromethane) could be used. A standard curve of α-tocopherol can then be used for standardization, which allows for the exact transfer of Trolox activity in the mixture of ethyl acetate and methanol.

Whenever possible, it is advisable to use both of the above methods to determine the antiradical activity of tested samples. The radicals used in them are not chemically identical. In the ABTS assay we used radical cations, which may facilitate the contact of some antioxidants dissociated into anions. They also differ in the preferred deactivation mechanism. Both systems accept and mix SET and HAT mechanisms, but ABTS radicals are more susceptible to the former, and DPPH to the latter (in the appropriate conditions). It is also possible to test this effect on the measured antioxidant activity, for example by modifying the pH of the buffer in the ABTS method or by changing the possibility of hydrogen transfer in the DPPH method. However, due to the comparability of research results from different authors, it is suggested to conduct the experiment also under standard conditions. Thus, the application of both methods will allow for a more comprehensive assessment of the tested compounds’ activity. The discovery of large differences in the results obtained may indicate the need for more detailed further research using specialized methods or methods simulating the real reaction in a more complex way.

The dependencies of the absorbance of non-deactivated radicals on the concentration of antioxidants are generally non-linear. Readings in any range of such dependence may lead to significant errors. Rapidly reacting antioxidants, such as Trolox or ascorbic acid, which are sometimes used as standards, yield linear relationships that can be misleading. If the IC_50_ parameter is not used (since basing on IC_50_ is more laborious) the range recommended in the literature (20–80% of the control absorbance) should be narrowed down to at least 30–70%. The lower limit results from non-linearities, the nature of which is usually more evident at higher antioxidant concentrations, and the upper limit results from the amplification of reading errors by calculations for more diluted samples. To ensure the possibility of a wide comparison of test results, a very good way to standardize the result is to use the calibration curve from Trolox solutions (α-tocopherol in a strictly hydrophobic environment) as a reference for the relative activity of the tested samples, read in the recommended range. Leaving the reading in the form of relative activity, depending not only on the reaction conditions but also on the dilution of the sample, eliminates the possibility of comparing the results with those of most publications.

## Figures and Tables

**Table 1:** Linear and non-linear relationships obtained for different antioxidant concentrations (μM) applied against ABTS radical cations in 50% acetone or methanol (radical solution diluted with ethanol) and buffers of pH 3.6 and 7.4 (radical solution diluted with buffers) after standard and modified reaction times. In case of linear relations, slope (a), correlation coefficient (r) and lower linearity limits if occurring (MIN–as % of A_contr_) were shown; in case of a non-linear relationship, equation type and correlation coefficient were shown.

Antioxidant	50% Acn + EtOH 6 min	50% Acn + EtOH 30 min	MeOH + EtOH 6 min	pH 3.6 6 min	pH 3.6 30 min	pH 7.4 6 min	pH 7.4 30 min
gallic acid	a = −98 r = −0.998	a = −109 r = −0.998	a = −97 r = −0.995	a = −69 r = −0.997	a = −85 r = −0.999	a = −112 −0.997	a = −125 −0.997
ferulic acid	power r = −0.996	power r = −0.997	power r = −0.998	N.A. ^1^	N.A.	N.A.	N.A.
catechin	power r = −0.995	a = −72 r = −0.991	power r = 0.997	N.A.	N.A.	N.A.	N.A.
quercetin	a = −78 r = −0.995 MIN = 30%	a = −92 r = −0.993	a = −78 r = −0.997 MIN = 35%	N.A.	N.A.	N.A.	N.A.
propyl gallate	a = −86 r = −0.999	a = −106 r = −0.999	a = −85 r = −0.999	a = −54 r = −0.996	a = −70 r = −0.998	a = −55 r = −0.998	a = −62 r = −0.997
glutathione	power r = −0.989	a = −57 r = −0.996 MIN = 35%	N.A.	power r = 0.999	power r = 0.993	a = −27 r = −0.995	a = −41 r = −0.996 MIN = 40%
carnosine	TR. ^2^	TR.	N.A.	N.D. ^3^	N.D.	N.D.	TR.
cysteine	a = −12 r = −0.995 MIN = 50%	a = −22 r = −0.986 MIN = 35%	N.A.	a = −12 r = −0.998	quadr. r = −0.996	a = −16 r = −0.993	power r = −0.977
histidine	N.D.	N.D.	N.A.	N.D.	N.D.	N.D.	N.D.
ascorbic acid	a = −30 r = −0.999	a = −28 r = −0.999	a = −30 r = −0.999	a = −26 r = −0.999	a = −26 r = −0.999	a = −28 r = −0.999	a = −27 r = −0.999
ascorbyl palmitate	N.A.	N.A.	a = −30 r = −0.999	N.A.	N.A.	N.A.	N.A.
α-tocopherol	N.A.	N.A.	a = −30 r = −0.999	N.A.	N.A.	N.A.	N.A.
tocopheryl acetate	N.A.	N.A.	N.D.	N.A.	N.A.	N.A.	N.A.
Trolox	a = −31 r = −0.999	a = −32 r = −0.999	a = −31 r = −0.999	a = −29 r = −0.999	a = −29 r = −0.999	a = −29 r = −0.999	a = −28 r = −0.999
phenolic mixture	a = −60 r = −0.988 MIN = 40%	a = −92 r = −0.995 MIN = 35%	a = −77 r = −0.997 MIN = 40%	N.A.	N.A.	N.A.	N.A.
peptide/AA mixture	a = −17 r = −0.994 MIN = 30%	a = −26 r = −0.999	N.A.	expon. r = −0.996	power r = −0.998	a = −10 r = −0.999	a = −15 r = −0.999
polar mixture	a = −43 r = −0.986 MIN = 40%	a = −50 r = −0.996	N.A.	a = −20 r = −0.999	a = −22 r = −0.993	a = −27 r = −0.999	a = −29 r = −0.999
applicable compounds mixture	N.A.	N.A.	a = −60 r = −0.997 MIN = 40%	N.A.	N.A.	N.A.	N.A.

^1^ N.A.—not applicable. ^2^ TR.—traces of activity. ^3^ N.D.—activity not detected.

**Table 2:** Linear and non-linear relationships obtained for different antioxidant concentrations (μM) applied against DPPH radicals in acetone or aqueous acetone after standard and modified reaction times (radical solution in methanol or acetone). In case of linear relations, slope (a), correlation coefficient (r) and lower/upper linearity limits if occurring (MIN/MAX–as % of A_contr_) were shown; in case of a non-linear relationship, equation type and correlation coefficient were shown.

Antioxidant	50% Acn + MeOH 10 min	50% Acn + MeOH 30 min	50% Acn + MeOH 120 min	70% Acn + MeOH 30 min	Acn + MeOH 30 min	Acn 30 min
gallic acid	a = −66 r = −0.994 MIN = 20%	a = −71 r = −0.999	a = −77 r = −0.997	a = −59 r = −0.994 MIN = 20%	expon. r = −0.996	expon. r = −0.996
ferulic acid	a = −12 r = −0.993 MIN = 40%	a = −14 r = −0.997 MIN = 30%	a = −15 r = −0.996 MIN = 20%	a = −11 r = −0.991 MIN = 30%	power r = −0.976	power r = −0.996
catechin	a = −20 r = −0.992 MIN = 15%	expon. r = −0.997	power r = 0.999	a = −19 r = −0.999 MIN = 10% MAX = 90%	a = −20 r = −0.999 MIN = 20%	expon. r = −0.999
quercetin	a = −35 r = −0.992 MIN = 30%	a = −57 r = −0.999	a = −57 r = −0.998	a = −56 r = −0.999 MIN = 20%	a = −34 r = −0.995 MIN = 40%	N.R. ^1^
propyl gallate	a = −46 r = −0.996	a = −54 r = −0.997	a = −60 r = −0.999	a = −53 r = −0.997 MIN = 15%	a = −48 r = −0.994 MIN = 35%	a = −39 r = −0.996 MIN = 25%
glutathione	power r = −0.993	power r = −0.989	power r = −0.996	expon. r = −0.992	N.A. ^2^	N.A.
carnosine	N.D. ^3^	TR. ^4^	TR.	N.A.	N.A.	N.A.
cysteine	a = −2 r = −0.993	a = −9 r = −0.998	a = −9 r = −0.997	N.A.	N.A.	N.A.
histidine	N.D.	TR.	TR.	N.A.	N.A.	N.A.
ascorbic acid	a = −22 r = −0.998 MIN = 10% MAX = 90%	a = −20 r = −0.995 MIN = 10% MAX = 90%	a = −21 r = −0.993 MIN = 10% MAX = 90%	a = −22 r = −0.998	N.A.	N.A.
ascorbyl palmitate	N.A.	N.A.	N.A.	a = −20 r = −0.997 MIN = 15%	a = −28 r = −0.995 MIN = 20% MAX = 90%	a = −29 r = −0.990 MIN = 25% MAX = 90%
α-tocopherol	N.A.	N.A.	N.A.	N.A.	a = −24 r = −0.999 MIN = 20%	a = −24 r = −0.999
tocopheryl acetate	N.A.	N.A.	N.A.	N.A.	TR.	TR.
Trolox	a = −24 r = −0.999	a = −26 r = −0.999	a = −24 r = −0.999	a = −25 r = −0.999	a = −24 r = −0.999 MIN = 20%	a = −24 r = −0.998
phenolic mixture	a = −32 r = −0.987	a = −36 r = −0.999 MIN = 20%	a = −37 r = −0.997 MIN = 30%	a = −40 r = −0.997 MIN = 30%	N.T. ^5^	N.T.
polar mixture	a = −20 r = −0.999 MIN = 20%	a = −20 r = −0.999 MIN = 20%	a = −22 r = −0.999 MIN = 30%	a = −22 r = −0.993 MIN = 20%	N.A.	N.A.

^1^ N.R.—no relationship between concentration and activity. ^2^ N.A.—not applicable. ^3^ N.D.—activity not detected. ^4^ TR.—traces of activity. ^5^ N.T.—not tested.

**Table 3:** Linear and non-linear relations obtained for different antioxidant concentrations (μM) applied against DPPH radicals in methanol or aqueous methanol after standard and modified reaction times (radical solution in methanol). In case of linear relations, slope (a), correlation coefficient (r) and lower/upper linearity limits if occurring (MIN/MAX–as % of A_contr_) were shown; in case of a non-linear relationship, equation type and correlation coefficient were shown.

Antioxidant	50% MeOH + MeOH 30 min	80% MeOH + MeOH 30 min	MeOH 10 min	MeOH 30 min	MeOH 120 min
gallic acid	a = −69 r = −0.997	a = −65 r = −0.996 MIN = 20%	a = −46 r = −0.996	a = −59 r = −0.996	a = −49 r = −0.996
ferulic acid	a = −12 r = −0.993 MIN = 40%	a = −12 r = −0.993 MIN = 40%	power r = −0.994	a = −14 r = −0.999 MIN = 50%	power r = −0.995
catechin	a = −23 r = −0.995 MIN = 20%	a = −27 r = −0.997 MIN = 10% MAX = 90%	a = −19 r = −0.994 MIN = 40%	a = −22 r = −0.998	a = −34 r = −0.996 MIN = 20%
quercetin	N.A. ^1^	a = −63 r = −0.999	a = −32 r = −0.999 MIN = 20%	a = −57 r = −0.999 MIN = 15%	a = −49 r = −0.998
propyl gallate	a = −52 r = −0.998	a = −53 r = −0.998	a = −47 r = −0.997	a = −63 r = −0.999	a = −47 r = −0.998
glutathione	power r = −0.996	N.A.	N.A.	N.A.	N.A.
carnosine	N.D. ^2^	N.A.	N.A.	N.A.	N.A.
cysteine	a = −12 r = −0.999	N.A.	N.A.	N.A.	N.A.
histidine	TR. ^3^	N.A.	N.A.	N.A.	N.A.
ascorbic acid	a = −22 r = −0.999 MIN = 10% MAX = 90%	a = −24 r = −0.999	a = −22 r = −0.995 MIN = 10% MAX = 90%	a = −29 r = −0.997 MIN = 20%	a = −22 r = −0.995
ascorbyl palmitate	N.A.	N.A.	a = −22 r = −0.999	a = −22 r = −0.998	a = −21 r = −0.998
α-tocopherol	N.A.	N.A.	a = −22 r = −0.999	a = −25 r = −0.998	a = −22 r = −0.999
tocopheryl acetate	N.A.	N.A.	N.D.	N.D.	N.D.
Trolox	a = −22 r = −0.999	a = −24 r = −0.999	a = −46 r = −0.999	a = −51 r = −0.999	a = −47 r = −0.993
phenolic mixture	a = −40 r = −0.995 MIN = 20%	N.T. ^4^	a = −22 r = −0.995 MIN = 25%	a = −24 r = −0.997 MIN = 20%	a = −26 r = −0.993
polar mixture	a = −27 r = −0.995 MIN = 20%	N.A.	N.A.	N.A.	N.A.
applicable compounds mixture	N.A.	N.A.	a = −19 r = −0.999 MIN = 20%	a = −20 r = −0.998 MIN = 20%	a = −25 r = −0.997 MIN = 30%

^1^ N.A.—not applicable. ^2^ N.D.—activity not detected. ^3^ TR.—traces of activity. ^4^ N.T.—not tested.

**Table 4:** Linear and non-linear relations obtained for different antioxidant concentrations (μM) applied against DPPH radicals in ethyl acetate (radical solution in methanol or ethyl acetate), dichloromethane (radical solution in methanol or dichloromethane) and hexane (radical solution in acetone or hexane) after standard reaction time. In case of linear relations, slope (a), correlation coefficient (r) and lower linearity limits if occurring (MIN–as % of A_contr_) were shown; in case of a non-linear relationship, equation type and correlation coefficient were shown.

Antioxidant	EtOAc + MeOH 30 min	EtOAc 30 min	DCM + MeOH 30 min	DCM 30 min	Hex + Acn 30 min	Hex 30 min
gallic acid	a = −57 r = 0.993 MIN = 25%	power r = −0.993	N.A. ^1^	N.A.	N.A.	N.A.
ferulic acid	expon. r = −0.993	power r = −0.993	N.A.	N.A.	N.A.	N.A.
catechin	a = −21 r = −0.999	expon. r = −0.998	N.A.	N.A.	N.A.	N.A.
quercetin	a = −33 r = −0.991 MIN = 35%	N.R. ^2^	N.A.	N.A.	N.A.	N.A.
propyl gallate	a = −50 r = −0.999	power r = −0.998	N.A.	N.A.	N.A.	N.A.
ascorbic acid	N.A.	N.A.	N.A.	N.A.	N.A.	N.A.
ascorbyl palmitate	a = −25 r = −0.998	a = −25 r = −0.999	N.A.	N.A.	N.A.	N.A.
α-tocopherol	a = −24 r = −0.998 MIN = 15%	a = −23 r = −0.999 MIN = 15%	a = −25 r = −0.999	a = −25 r = −0.999	a = −24 r = −0.999	a = −24 r = −0.999
tocopheryl acetate	N.D. ^3^	N.D.	N.D.	N.D.	TR. ^4^	N.D.
Trolox	a = −25 r = −0.999 MIN = 20%	a = −4 r = −0.999	N.A.	N.A.	N.A.	N.A.

^1^ N.A.—not applicable. ^2^ N.R.—no relationship between concentration and activity. ^3^ N.D.—activity not detected. ^4^ TR.—traces of activity.

**Table 5:** IC_50_ values (μM) determined against ABTS radical cations applied in 50% acetone or methanol (radical solution diluted with ethanol) and buffers of pH 3.6 and 7.4 (radical solution diluted with buffers) after standard and modified reaction times.

Antioxidant	50% Acn + EtOH 6 min	50% Acn + EtOH 30 min	MeOH + EtOH 6 min	pH 3.6 6 min	pH 3.6 30 min	pH 7.4 6 min	pH 7.4 30 min
gallic acid	3.38	2.95	3.53	4.81	3.95	2.93	2.57
ferulic acid	7.40	5.14	6.19	N.A. ^1^	N.A.	N.A.	N.A.
catechin	4.43	4.30	4.34	N.A.	N.A.	N.A.	N.A.
quercetin	4.05	3.39	3.95	N.A.	N.A.	N.A.	N.A.
propyl gallate	3.93	3.10	3.98	6.12	4.75	5.96	5.11
glutathione	10.47	5.39	N.A.	52.76	13.34	10.81	8.28
carnosine	V.N.D. ^2^	V.N.D.	N.A.	N.D. ^3^	N.D.	N.D.	V.N.D.
cysteine	31.36	11.73	N.A.	28.53	20.85	19.33	14.16
histidine	N.D.	N.D.	N.A.	N.D.	N.D.	N.D.	N.D.
ascorbic acid	11.59	11.82	11.73	13.57	13.53	12.33	12.62
ascorbyl palmitate	N.A.	N.A.	11.76	N.A.	N.A.	N.A.	N.A.
α-tocopherol	N.A.	N.A.	11.63	N.A.	N.A.	N.A.	N.A.
Trolox	11.09	10.59	11.10	12.07	12.21	11.98	12.24
phenolic mixture	5.97	3.46	4.51	N.A.	N.A.	N.A.	N.A.
peptide/AA mixture	17.23	12.75	N.A.	75.66	28.99	32.38	21.81
polar mixture	7.95	6.36	N.A.	15.93	15.35	12.46	11.27
applicable compounds mixture	N.A.	N.A.	5.79	N.A.	N.A.	N.A.	N.A.

^1^ N.A.—not applicable. ^2^ V.N.D.—value not determined. ^3^ N.D.—activity not detected.

**Table 6:** IC_50_ values (μM) determined against DPPH radicals applied in acetone or aqueous acetone after standard and modified reaction times (radical solution in methanol or acetone).

Antioxidant	50% Acn + MeOH 10 min	50% Acn + MeOH 30 min	50% Acn + MeOH 120 min	70% Acn + MeOH 30 min	Acn + MeOH 30 min	Acn 30 min
gallic acid	7.41	7.58	6.42	12.43	24.51	20.89
ferulic acid	42.04	33.65	30.81	77.83	221.09	1058.24
catechin	21.77	15.74	11.64	33.13	38.22	142.54
quercetin	11.35	9.31	8.83	31.50	26.79	V.N.D. ^1^
propyl gallate	10.62	9.79	8.49	13.99	15.78	19.53
glutathione	551.79	119.64	86.62	746.01	N.A. ^2^	N.A.
carnosine	N.D. ^3^	V.N.D.	V.N.D.	N.A.	N.A.	N.A.
cysteine	244.66	57.88	55.90	N.A.	N.A.	N.A.
histidine	N.D.	V.N.D.	V.N.D.	N.A.	N.A.	N.A.
ascorbic acid	29.96	30.04	29.69	39.69	N.A.	N.A.
ascorbyl palmitate	N.A.	N.A.	N.A.	42.85	32.15	36.48
α-tocopherol	N.A.	N.A.	N.A.	N.A.	33.77	35.98
Trolox	22.07	21.93	22.02	31.89	32.66	36.38
phenolic mixture	15.22	13.22	11.87	20.03	N.T. ^4^	N.T.
polar mixture	24.33	24.39	21.55	34.55	N.A.	N.A.

^1^ V.N.D.—value not determined. ^2^ N.A.—not applicable. ^3^ N.D.—activity not detected. ^4^ N.T.—not tested.

**Table 7:** IC_50_ values (μM) determined against DPPH radicals applied in methanol or aqueous methanol after standard and modified reaction times (radical solution in methanol).

Antioxidant	50% MeOH + MeOH 30 min	80% MeOH + MeOH 30 min	MeOH 10 min	MeOH 30 min	MeOH 120 min
gallic acid	7.29	10.69	10.90	9.21	9.61
ferulic acid	26.66	54.79	53.77	41.78	35.75
catechin	11.82	20.74	25.15	23.43	12.77
quercetin	N.A. ^1^	11.17	15.82	9.45	9.71
propyl gallate	9.38	13.46	11.36	10.40	10.08
glutathione	57.72	N.A.	N.A.	N.A.	N.A.
carnosine	N.D. ^2^	N.A.	N.A.	N.A.	N.A.
cysteine	46.39	N.A.	N.A.	N.A.	N.A.
histidine	V.N.D. ^3^	N.A.	N.A.	N.A.	N.A.
ascorbic acid	30.79	36.40	30.58	30.90	31.25
ascorbyl palmitate	N.A.	N.A.	24.02	23.60	21.89
α-tocopherol	N.A.	N.A.	23.25	22.56	21.11
Trolox	24.55	31.27	11.21	10.70	10.73
phenolic mixture	11.27	N.T. ^4^	22.13	21.23	16.17
polar mixture	16.96	N.A.	N.A.	N.A.	N.A.
applicable compounds mixture	N.A.	N.A.	24.92	24.27	18.62

^1^ N.A.—not applicable. ^2^ N.D.—activity not detected. ^3^ V.N.D.—value not determined. ^4^ N.T.—not tested.

**Table 8:** IC_50_ values (μM) determined against DPPH radicals applied in ethyl acetate (radical solution in methanol or ethyl acetate), dichloromethane (radical solution in methanol or dichloromethane) and hexane (radical solution in acetone or hexane) after standard reaction time.

Antioxidant	EtOAc + MeOH 30 min	EtOAc 30 min	DCM + MeOH 30 min	DCM 30 min	Hex + Acn 30 min	Hex 30 min
gallic acid	11.63	42.30	N.A. ^1^	N.A.	N.A.	N.A.
ferulic acid	198.87	1256.36	N.A.	N.A.	N.A.	N.A.
catechin	34.30	154.17	N.A.	N.A.	N.A.	N.A.
quercetin	22.80	V.N.D. ^2^	N.A.	N.A.	N.A.	N.A.
propyl gallate	14.73	35.85	N.A.	N.A.	N.A.	N.A.
ascorbyl palmitate	30.52	33.83	N.A.	N.A.	N.A.	N.A.
α-tocopherol	31.02	35.66	35.57	35.16	35.50	34.93
Trolox	30.88	235.08	N.A.	N.A.	N.A.	N.A.

^1^ N.A.—not applicable. ^2^ V.N.D.—value not determined.

**Table 9:** TEAC values determined against ABTS radical cations applied in 50% acetone or methanol (radical solution diluted with ethanol) after standard reaction time.

Antioxidant	50% Acn + EtOH 6 min			MeOH + EtOH 6 min		
	A = 0.2 × A_c_	A = 0.5 × A_c_	A = 0.8 × A_c_	A = 0.2 × A_c_	A = 0.5 × A_c_	A = 0.8 × A_c_
gallic acid		3.28			3.15	
ferulic acid	0.93	1.50	3.81	1.12	1.79	4.54
catechin	1.02	2.50	3.67	1.00	2.56	4.40
quercetin	2.29	2.74	3.20	1.99	2.81	3.21
propyl gallate		2.82			2.79	
glutathione	0.52	1.06	0.97		N.A. ^1^	
carnosine		~0.01			N.A.	
cysteine	0.24	0.35	0.45		N.A.	
histidine		N.D. ^2^			N.A.	
ascorbic acid		0.96			0.95	
ascorbyl palmitate		N.A.			0.94	
α-tocopherol		N.A.			0.95	
Trolox		1.00			1.00	
phenolic mixture	1.20	1.86	4.67	1.78	2.46	3.38
peptide/AA mixture	0.52	0.64	0.78		N.A.	
polar mixture	0.92	1.39	3.67		N.A.	
applicable compounds mixture		N.A.		1.48	1.92	2.79

^1^ N.A.—not applicable. ^2^ N.D.—activity not detected.

**Table 10:** TEAC values determined against ABTS radical cations applied in buffers (radical solution diluted with ethanol) after standard reaction time.

Antioxidant	pH 3.6 6 min			pH 7.4 6 min		
	A = 0.2 × A_c_	A = 0.5 × A_c_	A = 0.8 × A_c_	A = 0.2 × A_c_	A = 0.5 × A_c_	A = 0.8 × A_c_
gallic acid		2.51			4.09	
propyl gallate		1.97			2.01	
glutathione	0.09	0.23	0.38	0.94	1.11	1.23
carnosine		N.D. ^1^			N.D.	
cysteine		0.42		0.52	0.62	0.70
histidine		N.D.			N.D.	
ascorbic acid		0.89			0.97	
Trolox		1.00			1.00	
peptide/AA mixture	0.12	0.16	0.24		0.37	
polar mixture		0.80			0.96	

^1^ N.D.—activity not detected.

**Table 11:** TEAC values determined against DPPH radicals applied in aqueous acetone (radical solution in methanol) after standard reaction time.

Antioxidant	50% Acn + MeOH 30 min			70% Acn + MeOH 30 min		
	A = 0.2 × A_c_	A = 0.5 × A_c_	A = 0.8 × A_c_	A = 0.2 × A_c_	A = 0.5 × A_c_	A = 0.8 × A_c_
gallic acid		2.89		2.23	2.57	2.82
ferulic acid	0.45	0.65	0.80	0.29	0.41	0.51
catechin	0.95	1.39	1.83	0.83	0.96	1.07
quercetin		2.36		2.01	1.01	2.48
propyl gallate		2.24		2.01	2.28	2.44
glutathione	0.11	0.18	0.56	0.03	0.04	0.06
carnosine		~0.0007			N.A. ^1^	
cysteine		0.38			N.A.	
histidine		~0.0004			N.A.	
ascorbic acid		0.73 ^2^			0.80 ^2^	
Trolox		1.00			1.00	
phenolic mixture	1.50	1.66	1.91	1.44	1.59	1.84
polar mixture	0.83	0.90	1.08	0.85	0.92	1.10

^1^ N.A.—not applicable. ^2^ No non-linear relationship found.

**Table 12:** TEAC values determined against DPPH radicals applied in aqueous methanol (radical solution in methanol) after standard reaction time.

Antioxidant	50% MeOH + MeOH 30 min			80% MeOH + MeOH 30 min		
	A = 0.2 × A_c_	A = 0.5 × A_c_	A = 0.8 × A_c_	A = 0.2 × A_c_	A = 0.5 × A_c_	A = 0.8 × A_c_
gallic acid		3.37		2.53	2.93	3.23
ferulic acid	0.67	0.92	0.96	0.41	0.57	0.69
catechin	1.66	2.08	3.00	1.30	1.51	1.67
quercetin		N.A. ^1^			2.80	
propyl gallate		2.62			2.32	
glutathione	0.28	0.43	0.92		N.A.	
carnosine		N.D. ^2^			N.A.	
cysteine		0.53			N.A.	
histidine		~0.0002			N.A.	
ascorbic acid		0.80 ^3^			0.97	
Trolox		1.00			1.00	
phenolic mixture	1.89	2.18	2.73		N.T. ^4^	
polar mixture	1.34	1.45	1.60		N.A.	

^1^ N.A.—not applicable. ^2^ N.D.—activity not detected. ^3^ No non-linear relationship found. ^4^ N.T.—not tested.

**Table 13:** TEAC values determined against DPPH radicals applied in methanol or ethyl acetate (radical solution in methanol) after standard reaction time.

Antioxidant	MeOH + MeOH 30 min			EtOAc + MeOH 30 min		
	A = 0.2 × A_c_	A = 0.5 × A_c_	A = 0.8 × A_c_	A = 0.2 × A_c_	A = 0.5 × A_c_	A = 0.8 × A_c_
gallic acid		1.16		1.95	2.66	3.09
ferulic acid	0.17	0.26	0.36	0.11	0.16	0.20
catechin		0.46			0.90	
quercetin		1.13		1.16	1.35	1.51
propyl gallate		1.03			2.10	
ascorbic acid		0.35			N.A. ^1^	
ascorbyl palmitate		0.45			1.01	
α-tocopherol		0.47			1.00	
Trolox		1.00			1.00	
phenolic mixture		0.50			N.T. ^2^	
applicable compounds mixture	0.43	0.44	0.54		N.T.	

^1^ N.A.—not applicable. ^2^ N.T.—not tested.

## Data Availability

The data presented in this study are publicly available.

## Sample Availability

The samples are partly available.
